# Multidrug-Resistant *Acinetobacter baumannii* in Veterinary Clinics, Germany

**DOI:** 10.3201/eid1709.101931

**Published:** 2011-09

**Authors:** Sabrina Zordan, Ellen Prenger-Berninghoff, Reinhard Weiss, Tanny van der Reijden, Peterhans van den Broek, Georg Baljer, Lenie Dijkshoorn

**Affiliations:** Author affiliations: Justus-Liebig-University, Giessen, Germany (S. Zordan, E. Prenger-Berninghoff, R. Weiss, G. Baljer);; Leiden University Medical Center, Leiden, the Netherlands (T. van der Reijden, P. van den Broek, L. Dijkshoorn)

**Keywords:** zoonoses, Acinetobacter baumannii, animals, veterinary clinics, antimicrobial susceptibility, antimicrobial resistance, DNA fingerprinting, amplified fragment length polymorphism, pulsed-field gel electrophoresis, PFGE, clones, Germany, dispatch

## Abstract

An increase in prevalence of multidrug-resistant *Acinetobacter* spp. in hospitalized animals was observed at the Justus-Liebig-University (Germany). Genotypic analysis of 56 isolates during 2000–2008 showed 3 clusters that corresponded to European clones I–III. Results indicate spread of genotypically related strains within and among veterinary clinics in Germany.

Within the genus *Acinetobacter, A. baumannii* is clinically the most relevant species, frequently involved in hospital outbreaks and affecting critically ill humans (*1**,**2*). The strains involved are usually multidrug resistant, which limits therapeutic options (*3*). Many outbreaks in Europe and beyond have been associated with the European clones I–III (*4**–**6*).

Nosocomial infection in veterinary medicine is an emerging concern. The role of acinetobacters in diseases of hospitalized animals is largely unknown. Recent reports have documented occurrence of or infection with *Acinetobacter* spp., including *A. baumannii*, in hospitalized animals (*7**,**8*). The internal laboratory records of the microbiology department of the Giessen Veterinary Faculty (Institute for Hygiene and Infectious Diseases of Animals, Giessen, Germany) noted an increase in antimicrobial drug–resistant *Acinetobacter* isolates. To assess the species and type diversity of these organisms, we investigated a set of isolates from Giessen and other veterinary clinics obtained during a 9-year period by a combination of genotypic methods and compared the isolates for their susceptibility to antimicrobial drugs.

## The Study

The Institute for Hygiene and Infectious Diseases of Animals in Giessen receives samples for investigation from other veterinary departments of the university (mainly referral clinics) and from external veterinary clinics throughout Germany. During 2000–2008, *Acinetobacter* spp. were obtained from 137 hospitalized animals. From these animals, 56 isolates were selected for further characterization. The selection was made to reflect the diversity in epidemiologic origin of the collection regarding date of isolation, animal species, specimen, and veterinary clinic (82% from Giessen) (Table A1). Only isolates with possible clinical significance were included as inferred from the fact that they were the only or the dominating agent within the sample. Furthermore, according to data from the diagnostic laboratory, the selected isolates were highly resistant.

Confirmatory susceptibility testing of isolates was conducted by using the Clinical Laboratory Standards Institute broth dilution method (*9*) (Table). For precise species identification, amplified ribosomal DNA restriction analysis was performed. By this method, the 16S rDNA sequence was amplified by using PCR, followed by restriction of the amplified fragment by 5 restriction enzymes: *Cfo*I, *Alu*I, *Mbo*I, *Rsa*I, and *Msp*I. The combination of electrophoretic patterns of the respective enzymes was compared with a library of profiles (*10*).

**Table:** Resistance profiles of 56 animal *Acinetobacter* spp. isolates for 19 antimicrobial agents, obtained by CLSI broth microdilution test *

Profile; no. isolates	Tested antimicrobial agents																		
	Oxa	Pen	Ctn	Ery	Cli	Chl	Cst	Cvf	Amp	Amc	Tet	Enr	Orb	Dif	Kan	Sxt	Gen	Ipm	Amk
1; 1	R	R	R	R	R	R	R	R	R	R	R	R	I	R	R	R	R	R	S
2; 1	R	R	R	R	R	R	R	R	R	R	I	R	R	R	R	R	R	S	R
3; 28	R	R	R	R	R	R	R	R	R	R	R	R	R	R	R	R	R	S	S
4; 2	R	R	R	R	R	R	R	I	R	R	R	R	R	R	R	R	R	S	S
5; 2	R	R	R	R	R	R	R	R	R	I	R	R	R	R	R	R	R	S	S
6; 1	R	R	R	R	R	R	R	R	R	R	I	R	R	R	R	R	R	S	S
7; 1	R	R	R	R	R	R	R	R	R	R	R	R	R	R	S	R	R	S	S
8; 1	R	R	R	R	R	R	R	R	R	R	I	R	R	R	R	R	S	S	S
9; 3	R	R	R	R	R	R	R	R	R	R	R	R	R	R	R	S	S	S	S
10; 1	R	R	R	R	R	R	R	R	R	R	R	R	R	R	I	S	S	S	S
11; 1	R	R	R	R	R	R	R	R	R	R	I	R	R	R	R	S	S	S	S
12; 1	R	R	R	R	R	R	S	R	R	R	R	R	R	R	R	S	S	S	S
13; 1	R	R	R	R	R	R	R	R	R	I	R	S	S	S	R	R	R	S	S
14; 3	R	R	R	R	R	R	R	R	I	I	R	R	R	R	S	S	S	S	S
15; 1	R	R	R	R	R	R	R	R	I	I	R	R	R	R	S	S	S	S	S
16; 2	R	R	R	R	R	R	R	R	I	I	S	S	S	S	S	S	S	S	S
17; 1	R	R	R	R	R	R	R	R	I	S	S	S	S	S	S	S	S	S	S
18; 1	R	R	R	R	R	R	R	I	I	S	S	S	S	S	S	S	S	S	S
19; 1	R	R	R	R	R	R	R	R	S	S	S	S	S	S	S	S	S	S	S
20; 1	R	R	R	R	R	R	R	I	S	S	S	S	S	S	S	S	S	S	S
21; 1	R	R	R	R	R	R	S	R	S	S	S	S	S	S	S	S	S	S	S
22; 1	R	R	R	R	R	R	S	I	S	S	S	S	S	S	S	S	S	S	S

*CLSI guidelines M31-A2 (*9*). CLSI, Clinical Laboratory Standards Institute; Oxa, oxacillin; Pen, penicillin; Ctn, cephalotin; Ery, erythromycin; Cli, clindamycin; Chl, chloramphenicol; Cst, colistin; Cvf, cefovecin; Amp, ampicillin; Amc, amoxicillin/clavulanic acid; Tet, tetracycline; Enr, enrofloxacin; Orb, orbifloxacin; Dif, difloxacin; Kan, kanamycin; Sxt, trimethoprim/sulfamethoxazole; Gen, gentamicin; Ipm, imipenem; Amk, amikacin; R, resistant; I, intermediate; S, susceptible.

Fifty-two isolates were identified as belonging to *A. baumannii* and 3 to *A. pittii* (*Acinetobacter* gen. sp. 3) (*11*); 1 with a yet undescribed profile remained unclassified. Amplified fragment length polymorphism (AFLP) DNA fingerprint analysis was performed as described for confirmative species identification, for strain typing, and for clone identification (*4**,**12**,**13*). Briefly, *Eco*RI and *Mse*I were used to generate restriction fragments that were selectively amplified by using a Cy-5–labeled Eco-A and an Mse-C primer. Amplification products were separated by electrophoresis and subjected to cluster analysis with the BioNumerics software package 5.1 (Applied Maths, St-Martens-Latem, Belgium). For species identification, isolates were compared with reference strains of all described *Acinetobacter* species included in the Leiden University Medical Center AFLP database (Leiden, the Netherlands). Isolates with profiles >50% similar were considered to belong to the same species (*1*).

To assess the type diversity of the organisms, isolates were typed by pulsed-field gel electrophoresis (PFGE) (*14*) and by AFLP analysis. For PFGE, DNA was digested with the restriction endonuclease *Apa*I. Digitized profiles were analyzed with the BioNumerics software. For AFLP typing, a subset of 27 isolates was analyzed (Table A1). The profiles obtained were compared with each other and with those of the Leiden database, including those of the European clones I–III. A similarity cutoff level >80% was used to delineate members of the same clone and >90% to delineate organisms related at the strain level (*4**,**12**,**13*).

**Figure 1:**
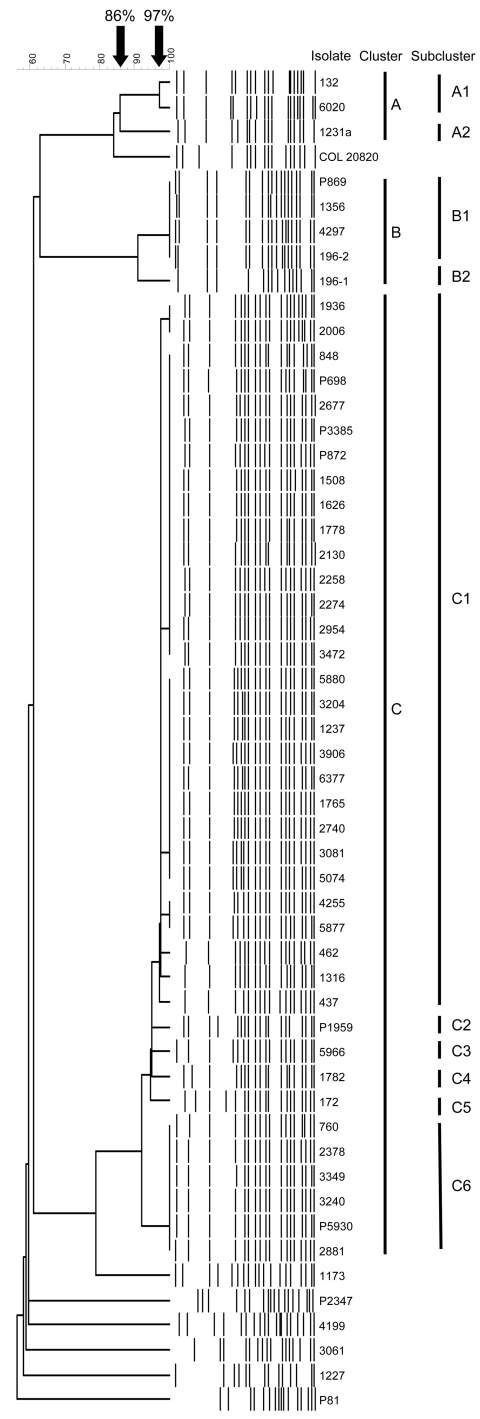
Computer-assisted cluster analysis of pulsed-field gel electrophoresis fingerprints of 53 *Acinetobacter* *baumannii* and 2 *Acinetobacter* spp. pittii isolates. COL 20820 was used as the reference standard for normalization of the digitized gels (*14*).

For PFGE, at a similarity level of 86%, 3 major clusters (A, B, and C) and 6 unique isolates were distinguished (Figure 1). Within major cluster C, 2 main subclusters (C1 and C6) and 4 single profiles (C2–C5) were observed at 97% similarity (Table A1; Figure 1). Despite some band differences, the patterns in major cluster C were strikingly similar. The maximum number of band differences in subcluster C1 was 3, which indicates that the organisms were genetically closely related. In subcluster C6, only minor differences in size of the fragments were observed (Figure 1).

**Figure 2:**
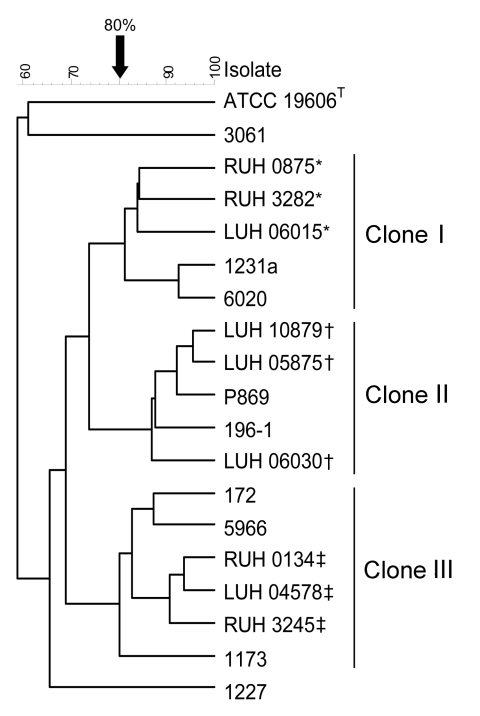
Amplified fragment length polymorphism analysis of 9 animal *Acinetobacter baumannii* isolates belonging to the major pulsed-field gel electrophoresis types and 9 reference strains of the European clones I–III from the Leiden University Medical Center collection. *Reference strains of European clone I; †reference strains of European clone III; ‡reference strains of European clone II.

For AFLP, we investigated a subset of 27 isolates, including at least 1 isolate of each of the 16 different PFGE profiles and the 3 isolates nontypeable by PFGE. Seventeen AFLP types were distinguished at the 90% similarity cutoff level for strain delineation. Identification by AFLP showed full agreement with amplified ribosomal DNA restriction analysis species identification (Table A1). Comparison of isolates to those of the Leiden AFLP database grouped isolates with AFLP profile 8 (corresponding PFGE profiles A1, A2) with isolates of European clone I, those with profiles 10–16 (corresponding PFGE profile C1–C6) with clone II, and with profile 7 (corresponding PFGE profiles B1, B2) with clone III (Table A1). Examples are shown in Figure 2.

## Conclusions

The occurrence of PFGE type C in different animals admitted to 3 different clinical wards of the Justus-Liebig-University Giessen over 9 years might indicate endemic occurrence of these organisms on these wards. Survival in the hospital environment (*15*), patient-to-patient transfer, and transfer from 1 animal clinic to another may have contributed to their persistence and spread. Because veterinarians, stockmen, and students rotate between the various clinics and departments, transmission by hands or equipment should be considered. Frequent transport of colonized animals to and from shared examination rooms, e.g., for computer-assisted tomography, might also have contributed to the chain of spread. Because type C isolates also were found in samples from animal clinics throughout Germany (Table A1), limited genetic variation in animal strains of *A. baumannii* also is possible.

AFLP data were, further to comparative typing of the animal isolates, also used to assess the relatedness of the isolates in our study to those of the widespread European clones I–III that represent genetically related but not identical strains that are frequently multidrug resistant and associated with epidemic spread in human clinics (*1**,**4**–**6*). Although not all strains were characterized by AFLP, we conclude by inductive generalization of results that the findings apply to all isolates of the PFGE types from which the organisms were selected. Thus, a large proportion of the animal *A. baumannii* isolates were genetically congruent with the European clone I, II, or III. Occurrence of such isolates in ill, hospitalized animals of various species might indicate that, as in human medicine, *A. baumannii* is an emerging opportunistic pathogen in veterinary medicine. The occurrence of clones I–III in animals and humans also raises concern about whether the organisms can spread from animals to humans or whether the animals have acquired the organisms from humans.

The occurrence of genotypically related, antimicrobial drug–resistant *A. baumannii* strains in hospitalized animals suggests that these organisms are most likely nosocomial pathogens for animals. If so, veterinary clinics face a great challenge regarding prevention, control, and treatment of infections with these organisms, similar to situations in human hospitals. Finally, the possibility of spread from humans to animals or vice versa requires special attention.

## Appendix

**Table A1:** Origin and drug resistance profiles of *Acinetobacter* spp. isolates collected from veterinary specimens from Germany, 2000–2008*

Isolate	Species†	Isolation date	City	Clinic	Animal	Specimen	PFGE cluster	AFLP type	European clone‡	Resistance profile§
5880	*baum.*	2000 Nov 22	Giessen	MVK	Dog	Feces	C1	ND	ND	3
5877¶	*baum.*	2000 Nov 22	Giessen	MVK	Cat	Urine	C1	10	II	3
5966¶	*baum.*	2000 Nov 24	Giessen	CVK	Cat	Urine	C3	10	II	9
6020¶	*baum.*	2000 Nov 28	Giessen	MVK	Cat	Urine	A1	8	I	1
6377	*baum.*	2000 Dec 13	Giessen	CVK	Cat	Urine	C1	ND	ND	3
132	*baum.*	2001 Jan 9	Giessen	MVK	Dog	Pericardium	A1	ND	ND	13
1237	*baum.*	2001 Mar 12	Giessen	CVK	Dog	Urine	C1	ND	ND	3
1765	*baum.*	2001 Apr 5	Giessen	CVK	Cat	Urine	C1	ND	ND	3
2740¶	*baum.*	2001 May 31	Giessen	CVK	Cat	Urine	C1	10	II	3
3906	*baum.*	2001 Aug 16	Giessen	CVK	Dog	Urine	C1	ND	ND	3
4255	*baum.*	2001 Aug 28	Giessen	CVK	Dog	Abscess	C1	ND	ND	3
5074	*baum.*	2001 Oct 3	Giessen	MVK	Dog	Wound	C1	ND	ND	3
3204¶	*baum.*	2002 Feb 1	Giessen	CVK	Horse	Tendon	C1	11	II	3
P1697¶	*baum.*	2002 Mar 7	Bad Marienberg	Private	Horse	Uterus	NT	6	NA	17
1508	*baum.*	2002 Apr 2	Giessen	AGVK	Cat	Urine	C1	ND	ND	3
1626	*baum.*	2002 Apr 9	Giessen	CVK	Cat	Urine	C1	ND	ND	3
1782¶	*baum.*	2002 Apr 16	Giessen	CVK	Cat	Urine	C4	11	II	3
1778	*baum.*	2002 Apr 16	Giessen	CVK	Cat	Urine	C1	ND	ND	3
P3385	*baum.*	2002 Apr 30	Frankfurt/M.	Private	Dog	Bronchia	C1	ND	ND	4
2258¶	*baum.*	2002 May 14	Giessen	CVK	Cat	Urine	C1	13	II	3
2274	*baum.*	2002 May 15	Giessen	CVK	Cat	Urine	C1	ND	ND	4
2378	*baum.*	2002 May 17	Giessen	CVK	Dog	Subcutis	C6	ND	ND	15
2881	*baum.*	2002 Jun 12	Giessen	CVK	Cat	Urine	C6	ND	ND	14
2954	*baum.*	2002 Jun 14	Giessen	CVK	Cat	Urine	C1	ND	ND	3
3081	*baum.*	2002 Jun 24	Giessen	CVK	Cat	Urine	C1	ND	ND	3
3240	*baum.*	2002 Jul 3	Giessen	MVK	Dog	Feces	C6	ND	ND	10
3349	*baum.*	2002 Jul 10	Giessen	MVK	Dog	Duodenum	C6	ND	ND	14
3472	*baum.*	2002 Jul 18	Giessen	AGVK	Dog	Vagina	C1	ND	ND	3
P5930	*baum.*	2002 Aug 18	Betzdorf	Private	Dog	Serum	C6	ND	ND	14
P81¶	gen. sp.3	2003 Jan 8	Duisburg	Private	Dog	Nose	Un	1	ND	20
172¶	*baum.*	2003 Jan 14	Giessen	CVK	Dog	Urine	C5	13	II	5
P872	*baum.*	2003 Feb 8	Duisberg	Private	Dog	Nose	C1	ND	ND	3
848	*baum.*	2003 Feb 26	Giessen	MVK	Dog	Bronchia	C1	ND	ND	3
2130	*baum.*	2003 Mar 19	Giessen	Pathology	Cat	Urine	C1	ND	ND	5
P1959¶	*baum.*	2003 Mar 20	Heidelberg	Private	Cat	Thorax	C2	12	II	7
2677¶	*baum.*	2003 May 26	Giessen	MVK	Dog	Urine	C1	14	II	3
4297	*baum.*	2005 Dec 15	Giessen	CVK	Dog	Wound	B1	ND	ND	11
196–1¶	*baum.*	2006 Jan 19	Giessen	MVK	Cat	Urine	B2	7	III	8
196–2	*baum.*	2006 Jan 19	Giessen	MVK	Cat	Urine	B1	ND	ND	12
437¶	*baum.*	2006 Jan 31	Giessen	CVK	Cat	Ear	C1	13	II	9
462¶	*baum.*	2006 Feb 1	Giessen	MVK	NR	Exam table	C1	13	II	3
P698	*baum.*	2006 Feb 4	Hofheim	Private	Cat	Urine	C1	ND	ND	3
580¶	NC	2006 Feb 9	Giessen	CVK	Dog	Nose	NT	4	–	16
1231a¶	*baum.*	2006 Mar 24	Giessen	MVK	Cat	Urine	A2	8	I	3
1316¶	*baum.*	2006 Apr 3	Giessen	CVK	Cat	Urine	C1	13	II	3
P869¶	*baum.*	2008 Feb 1	Nauen	Private	Horse	Cervix	B1	7	III	2
760¶	*baum.*	2008 Feb 2	Giessen	MVK	Dog	Blood	C6	13	II	9
1173¶	*baum.*	2008 Feb 13	Giessen	CVK	Dog	Wound	Un	16	II	3
1227¶	*baum.*	2008 Feb 18	Giessen	AGVK	Dog	Vagina	Un	9	NA	19
1356	*baum.*	2008 Feb 21	Giessen	CVK	Dog	Fistula	B1	ND	ND	6
P2134–1¶	gen. sp.3	2008 Mar 20	Solingen	Private	Bird	Pharynx	NT	2	ND	22
P2347¶	gen. sp.3	2008 Mar 29	Dortmund	Private	Guinea pig	Lips	Un	3	ND	18
1936¶	*baum.*	2008 Mar 29	Giessen	MVK	Dog	CVC	C1	15	II	3
2006	*baum.*	2008 Apr 3	Giessen	MVK	Dog	CVC	C1	ND	ND	3
3061¶	*baum.*	2006 Jun 12	Giessen	AGVK	Cow	Udder	Un	17	NA	21
4199¶	*baum.*	2008 Aug 15	Giessen	CVK	Dog	Vagina	Un	5	NA	16

*PFGE, pulsed-field gel electrophoresis; AFLP, amplified fragment-length polymorphism; *baum*., *baumannii*; MVK, medical department, small animal clinic of the Justus-Liebig-University Giessen; ND, not done; CVK, surgical department, small animal clinic of the Justus-Liebig-University Giessen; NT, nontypeable; NA, not affiliated with either of the European clones I–III; AGVK, gynecologic and andrologic department of the Justus-Liebig-University Giessen; gen. sp., genomic species; NR, not relevant; NC, not classified; CVC, central venous catheter; Un, unique PFGE strain (strain does not belong to either PFGE cluster A, B, or C, indicating a low degree of genetic related). †Taxonomic designation according to ARDRA results, AFLP being complementary. Species, Acinetobacter species. ‡European clones as described (*5**,**6*) and delineated by AFLP at ≈80% similarity cutoff level (*4*). §Profile as given in Table. ¶Isolates tested by AFLP.
